# Experimental evidence for reciprocity in allogrooming among wild-type Norway rats

**DOI:** 10.1038/s41598-017-03841-3

**Published:** 2017-06-21

**Authors:** Manon K. Schweinfurth, Binia Stieger, Michael Taborsky

**Affiliations:** Institute of Ecology and Evolution, University of Bern, Wohlenstr. 50a, CH-3032 Hinterkappelen, Switzerland

## Abstract

If individuals help more those who have previously helped them, stable cooperation may ensue through alternation of roles between donors and recipients. Allogrooming, which is costly to donors and beneficial to recipients, is often exchanged between social partners. Arguably, allogrooming and allopreening are the most frequently exchanged social services and have been used as a standard model of reciprocal cooperation. However, evidence for the application of reciprocity rules among social partners allogrooming each other hitherto is merely correlational. Here, we tested whether female Norway rats (*Rattus norvegicus*) apply the decision rule characterising direct reciprocity: help someone who has helped you before, by experimentally manipulating both the need for allogrooming and the behavioural response. Furthermore, we checked whether trading of grooming services is influenced by the rank of the social partner. We show that rats groom social partners reciprocally and prefer to do so up the hierarchy, i.e. they groom dominant partners more often than subordinates, while reciprocating with both. This provides experimental evidence that animals render a costly social service by applying reciprocity decision rules when showing a natural hygienic behaviour. The fact that allogrooming is more readily shown up the hierarchy may suggest an appeasing function.

## Introduction

Allogrooming, the grooming of one animal by another of the same species by licking or carefully nibbling, is common in animals ranging from arthropods to apes^1^. Allogrooming and allopreening can be altruistic behaviours providing benefits to a recipient at a cost to the donor. Potential benefits include the removal of ectoparasites or debris^2^, and the spread of antimicrobial substances^3^. Potential costs to donors include the loss of saliva and electrolytes^4^ and opportunity costs due to investment in time and energy^4–8^. Besides caring for the body surface of social partners, allogrooming may play a crucial role in social relationships. It can help to provide access to mating partners^9^, increase tolerance between social partners^10^, reduce aggression^11^ and induce agonistic support between grooming partners^12^.

Nevertheless, the underlying behavioural and evolutionary mechanisms of this cooperative behaviour are still poorly understood. Possible mechanisms responsible for this behaviour include coercion^13^, kin selection^14^ and reciprocity^15^. Allogrooming often reflects a reciprocal exchange of given and received grooming bouts between social partners (e.g.: impala^16^, herb-field mouse^17^, chimpanzee^18^; reviewed in ref. 19): by alternating the roles between donor and recipient, the payoffs of social partners can correlate among one another and reciprocal cooperation becomes stable over time^19, 20^.

Despite its widespread occurrence, hitherto the evidence for reciprocity in allogrooming has been based exclusively on correlational data. To demonstrate the application of reciprocity rules by animals, a correlation between grooming given and received is not sufficient. Confounding effects such as bonds^21^, relatedness^22^ or spatial proximity^23^ may also explain this correlation. Furthermore, reciprocal trading between different social services^24^ increases the variance between given and received favours and therefore may decrease the likelihood of finding reciprocal exchanges within one commodity. Experimental evidence based on manipulation of behaviour is required to disentangle underlying mechanisms from confounding factors.

When social services are traded reciprocally, social rank may also affect the outcome of interactions among conspecifics^25^. Seyfarth’s (1977) theoretical model predicts that higher ranked individuals receive more grooming than lower ranked partners, and grooming bouts should be unreciprocated if directed up the hierarchy (for a critical review of the model see ref. 26). However, empirical studies provide conflicting results. A meta-analysis using 14 primate species provides support for the model^27^, whereas other authors report allogrooming down a hierarchy or a similar exchange regardless of relative rank^28, 29^. Most research has focused on social primates, which is why little is known about the explanatory power of the model outside of primates. In line with Seyfarth’s (1977) model, meerkats have a pronounced hierarchy and show allogrooming as an appeasement behaviour up the hierarchy^30^. In contrast, badgers that also have a pronounced hierarchy groom partners based on short-term reciprocal exchanges, regardless of dominance relationships^31^. Green woodhoopoes diverge in their allopreening behaviour according to different body parts^32^. Whereas spots that are difficult to reach are allopreened reciprocally independent of rank, allopreening of the rest of the body is directed more often to dominants, and allopreening initiated by dominants is more often reciprocated than if it is initiated by subordinates. These non-primate examples illustrate that reciprocal exchange of hygienic care may be highly specific to the context and the hierarchical structure of a social system.

To test experimentally whether animals apply reciprocity decision rules such as “help someone who has helped you”, and to check for a potential influence of hierarchical relationships on reciprocal exchange of social services, we manipulated allogrooming behaviour in wild-type Norway rats. In this experiment we induced a need for allogrooming among social partners and created cooperative and non-cooperative individuals by allowing or preventing allogrooming. To our knowledge, this is the first study investigating the causal relationship between cooperation received and cooperation given in the context of social hygiene. Our model organism is wild-type Norway rats (*Rattus norvegicus*), which are highly social animals living in colonies of various sizes^33^. Under natural conditions, they show a range of affiliative behaviours, such as allogrooming, huddling and food sharing^34^. Rats have a social hierarchy^35^, show a high propensity to cooperate^36^ and share food with each other according to generalized^37, 38^ and direct reciprocity^38–41^.

We predicted that if allogrooming is exchanged reciprocally, rats would more often groom those partners that had previously groomed them. According to conflicting results from previous theoretical and empirical work, we predicted several outcomes regarding the influence of hierarchical relationships on allogrooming. If allogrooming is directed up the hierarchy, we expected female rats to groom dominant partners more often than subordinates, independently of previously experienced grooming levels. We expected the opposite effect if allogrooming in rats primarily serves as suppression. Furthermore, an interaction between the two potential causal mechanisms is conceivable as well. Previously experienced allogrooming received from dominant or subordinate partners might be reciprocated only with one partner.

## Materials and Methods

### Experimental subjects and holding conditions

We used adult female Norway rats, which were outbred offspring of wild rats (source: Animal Physiology Department, University of Groningen, Netherlands). Individuals weighed approximately 350 g. The rats were habituated to handling right after weaning and hence did not show any signs of stress when being handled, transported to the experimental room and exposed to the setup and an observer. They were individually marked by ear punches and were housed with littermates in groups of five^42^. In accordance with animal welfare legislation of Switzerland (Tierschutzverordnung Schweiz 04/2008), we enriched the cages (80/50/37.5 cm) with various materials a wooden house and board, a tunnel, a piece of wood to nibble, a cardboard roll to play, digging-material (wood shavings), nest-building material (hay) and a salt block^43^. Food (conventional rat pellets) and water were provided *ad libitum*, except one food deprivation before the hierarchy test, as described below. In addition to *ad libitum* food, rats received once a day a corn mix, fresh fruits or vegetables. The ambient temperature was 20 °C ± 1 °C, with a relative humidity of 50–60% and a 12:12 h light/dark cycle with lights on at 20:00 hours, and 30 minutes of dawn and dusk. As rats are nocturnal, all tests were conducted during the dark phase under red light^44^. Animal housing and experimentation were approved by and conducted in accordance with the guidelines set by the Swiss Federal Veterinary Office under license BE25/14.

### Hierarchy assessment

To determine the hierarchical structure of each group, we conducted a food-allocation test (modified after ref. 45) with all present rats (pairwise trials with 55 rats housed in 11 groups). We placed a high-value food item (piece of banana) covered with a perforated plastic cup in the middle of an experimental arena (80/40/40 cm). Two out of five rats of each group were then placed in the arena. After the plastic cup was explored by both individuals, we removed the rats and uncovered the food item. Directly after uncovering the food item, we again placed the two cage mates in the arena, both at the same distance to the available food item. The winner was defined as the rat monopolizing the treat by either gaining or keeping the food item. In cases of uncertainty, the test was repeated. Each rat was tested against all four littermates. We increased the motivation level of both partners to gain the food reward by prior food deprivation for at most 10 hours during the inactive phase of the rats’ daily cycle (whereas water access was provided *ad libitum*).

This food-allocation test allowed us to determine the most dominant and subordinate rat of each group. As shown by a repeatability analysis, ranks in female Norway rats are consistent over time^46^. Two out of the three intermediate rats of each group were chosen randomly to become the focal rats in the experiment, which were tested with both types of social partners, the most dominant and the most subordinate individual of each group.

### Experiment

**Figure 1:**
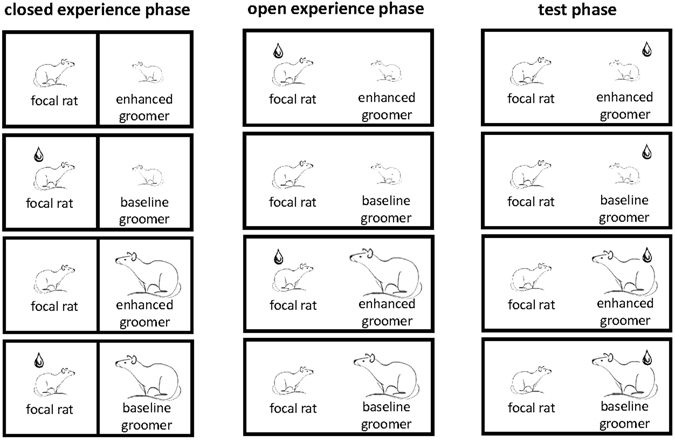
Test procedure. Every focal rat experienced all four treatments (lines from top to bottom) in a randomized order. In the example shown, the focal rat first experienced a subordinate partner (depicted small in graph) as being a cooperative groomer (=grooming at an enhanced level), by applying saltwater on the focal rat’s neck during the open experience phase, in which the partner had the possibility to allogroom the focal rat (first line). The same partner was experienced as being non-cooperative when we applied saltwater during the closed experience phase (second line), in which the partners were physically prevented from allogrooming by a separating mesh. We repeated the same treatments with a dominant cage mate (depicted large in graph; lines 3 and 4). We recorded during the test phase when and how often focal individuals groomed the previously experienced partner, which was now treated with saltwater to induce allogrooming of the focal individual. Drawings were kindly provided by Valentina Balzarini.

Focal rats (*n* = 22) were tested in four different treatments with two social partners each (Fig. 1). Each focal rat was first exposed to the experience phase, which consisted of two phases where they experienced either a cooperative or a non-cooperative interaction with a dominant or a subordinate partner. Each experience phase, consisting of a closed and open phase, was directly followed by a test phase, in which we recorded the allogrooming behaviour of the focal individual towards the same social partner.

We experimentally induced allogrooming between social partners by an application of saltwater (250 g salt/l) onto the focal rat’s neck during the experience phase. We chose saltwater in contrast to some preferred fluid because rats show aversion to salt^47^ (SI Fig. 1 for data on non-preferred saltwater concentration), in order to exclude that rats would merely lick the liquid off a partner to their own pleasure or benefit. The saltwater was applied with a cotton stick to the neck of the rat (seven times streaking). The neck was used because it cannot be easily autogroomed, and in a pilot study we found that most allogrooming is directed towards the neck of partners.

To manipulate the allogrooming experience of focal rats, the experience phase consisted of two stages: in one of them, physical interactions and allogrooming were prevented by a separating wire mesh (‘closed experience phase’). In the other one, the dividing mesh was removed and the rats could freely interact (‘open experience phase’). Both conditions were further split up into two different treatments: in the ‘cooperative allogrooming treatment’, focal individuals received a saltwater application shortly before exposure to a partner that could allogroom them (i.e., without dividing mesh), whereas in the ‘non-cooperative allogrooming treatment’, focal individuals received a saltwater application shortly before exposure to a partner that was prevented to allogroom them by a divider. By splitting up the experience phase into two phases, focal rats always received a saltwater application but only when saltwater was applied in the open experience phase, the partner could allogroom, which lead to more allogrooming. After the focal rats experienced the partner as cooperative or non-cooperative allogroomers, we conducted the test phase, in which only the respective social partner was treated with a saltwater application. The open and close experience phase and the test phase lasted for 20 min each and took place in the same arena as the hierarchy assessment. We video-recorded all three phases (camera: Sony: HDR-CX550) and analysed the videos using the program Solomon Coder (version: beta 14.03.10). All allogrooming events were counted, and the latency to the first grooming event of each partner was measured. An allogrooming bout was defined as one individual repeatedly nibbling and licking the body surface of the other except the anal region. A new event was recorded, if the allogrooming had been interrupted for at least 10 sec. As most grooming events were short (median bout duration: 2.85 sec), we chose to analyse the frequency instead of the duration of allogrooming.

The test order of focal individuals was randomly chosen. We randomized the order of all four treatments (dominant cooperative allogrooming, dominant non-cooperative allogrooming, subordinate cooperative allogrooming, and subordinate non-cooperative allogrooming) for all individuals and prevented treatments from being tested more often than others on a particular day. We also randomized the order of the two phases of the experience phase, ensuring that every rat experienced each order twice, once with and once without cooperative allogrooming experience coming first. Every focal rat experienced only one treatment per day and we kept the time of day constant for every rat over all trials. We conducted the experiment on consecutive days. All randomizations were done in Microsoft Excel using the command RAND.

### Statistical analyses

To check whether the experienced grooming frequency of focal rats differed between the cooperative and non-cooperative allogrooming treatments, we compared the experienced allogrooming frequencies between the open experience phases with and without saltwater application with a Wilcoxon matched-pairs signed-ranks test. We used a non-parametric test because allogrooming rates were not normally distributed (Shapiro-Wilk test: W = 0.95, *p* = 0.002).

To test for reciprocal grooming between focal rats and their social partners, we used a generalized linear mixed effects model (GLMM). We included the focal rats’ frequency of given grooms in the test phase as dependent variable, cooperation level (=cooperative vs. non-cooperative experience) and hierarchy (=dominant vs. subordinate partner) as fixed factors, and rat identity and housing group as random effects. We simplified our model by removing the non-significant interaction between cooperation level and hierarchy^48^. In addition, we tested whether received and given allogrooming bouts correlate by using a generalized linear mixed model. We included focal rats’ given allogrooming bouts as dependent variable and the experienced allogrooming bouts during the open experience phase as explanatory variable. Because focal rats were tested repeatedly, we again included rat identity and housing group as random effects. We checked in both models for overdispersion of data, which was not the case. We used generalized models because allogrooming rates were not normally distributed (Shapiro-Wilk test: W = 0.94, *p* < 0.001) and hence assumed a Poisson distribution with a log link function.

To test for differences in the focal rats’ latency to allogroom their social partner, we conducted a survival analysis with the latencies as the dependent variable, cooperation level and hierarchy as fixed factors, and rat identity as a random factor. Again we simplified the model by removing the non-significant interaction between treatment and hierarchy. We analysed the data with R (Version: 3.1.0.) using the packages: lme4, car and survival.

## Results

**Figure 2:**
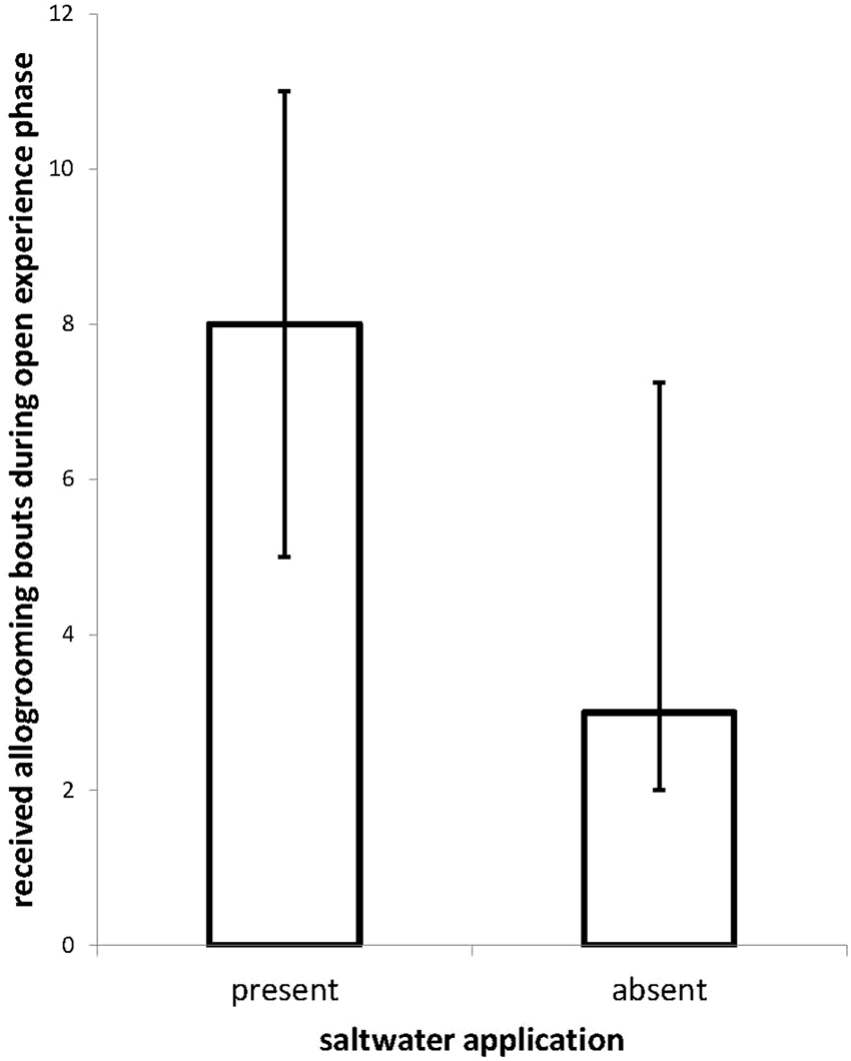
Experience phase. Focal rats received more grooming bouts during the open experience phase in which their partners could freely interact with them after saltwater had been applied onto their neck compared to when no saltwater had been applied in this phase. Median grooming frequencies ± interquartile ranges are depicted per 20 min observation period.

Social partners allogroomed focal rats more often in the open experience phase in which saltwater was applied compared to when no saltwater was applied (effect size: 3 allogrooming bouts; Wilcoxon: V = 165.5, n = 22, p < 0.001; Fig. 2). This confirms that with our treatment, the grooming frequencies of social partners were successfully manipulated.

**Figure 3:**
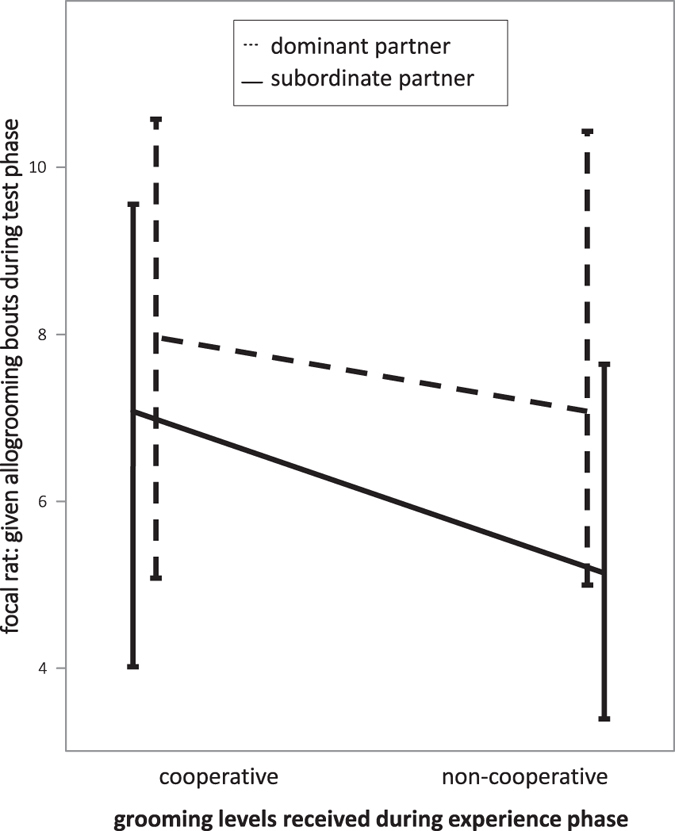
Grooming bouts of focal rats depending on their partner’s previous cooperation level and relative rank. Focal rats more often groomed those partners that had previously groomed them after saltwater had been applied (cooperation experience) than those that had been prevented to groom them in this situation (non-cooperative experience; cf. Fig. 1). Dominant partners overall received more grooming bouts than subordinate individuals, and there was no interaction between the effects of rank and of previous cooperation experience. Median grooming frequencies ± interquartile ranges are shown by focal rats during the test phase per 20 min observational period.

When focal rats had experienced a cooperative allogrooming partner, they groomed this partner significantly more often in the test phase than after experiencing a non-cooperative allogrooming partner (GLMM, ß = −0.19 ± 0.08, X^2^ = 5.96, *p* = 0.015; Fig. 3). Furthermore, focal rats more often allogroomed dominant than subordinate partners (GLMM, ß = −0.17 ± 0.08, X^2^ = 4.52, *p* = 0.033; Fig. 3). Focal individuals reciprocated grooming bouts with both dominant and subordinate partners, as there was no interaction effect between the partner’s allogrooming level and rank (GLMM, ß = −0.14 ± 0.15, X^2^ = 0.84, *p* = 0.36; Fig. 1; SI Figs 2 and 3). Allogrooming rates provided by focal rats did not significantly correlate with allogrooming rates received from their respective partners (GLMM, ß = 0.01 ± 0.01, X^2^ = 0.86, *p* = 0.35).

Neither the previously experienced allogrooming level of the partner (Proportional Hazards Regression Model: ß = −0.20 ± 0.22, X^2^ = 0.84, *p* = 0.36) nor the relative rank (Proportional Hazards Regression Model: ß = −0.14 ± 0.22, X^2^ = 0.38, *p* = 0.54) influenced the latency to the first grooming event during the test phase (SI Fig. 4).

## Discussion

In this study, we established a novel method to manipulate allogrooming behaviour, which was achieved by the application of saltwater to a part of the body that cannot be reached easily by autogrooming. This allowed us to test whether allogrooming is exchanged reciprocally among experimental subjects. Our data show that Norway rats indeed apply reciprocity rules when allogrooming each other. Received allogrooming consistently raised the propensity of rats to return this favour to their social partner. Further, we tested whether rats modify their grooming service according to their partner’s rank. Received grooming was reciprocated to both dominant and subordinate partners, but rats groomed dominant partners generally more often than subordinate partners. This suggests that reciprocal trading is important when allogrooming is directed both to higher and lower ranked partners, but higher ranked rats generally receive more allogrooming.

Reciprocal allogrooming has been argued to be “a low-cost form of reciprocity involving low energy expenditure and risk, because if a bout is not reciprocated, the initiator has lost little”^49, 50^. However, significant costs of grooming and preening due to time and energy expenditure and reduced vigilance have been demonstrated in a range of animals (reviewed in ref. 19) and when animals allogroom conspecifics they may increase the probability of parasite transmission^51^. Here we show that rats accept the costs of allogrooming partners against their preference, because saltwater, as applied in our experiment, is usually avoided by them. Even without the influence of salt on the water balance of rats, grooming causes loss of saliva, i.e. water^4^. Hence under natural conditions, the allogrooming behaviour shown by our experimental subjects might entail fitness costs (cf. ref. 19).

In accordance with food sharing^39^, allogrooming is another social service that is exchanged reciprocally in female Norway rats. However, we cannot determine from the current experiment whether rats groom conspecifics according to direct or generalized reciprocity rules^37, 52^. Direct reciprocity is partner specific and follows the rule “help someone who has helped you”. Generalized reciprocity corresponds to a cognitively much simpler rule of thumb “help anyone if helped by someone”, which can establish stable cooperation in various conditions and even in large groups^53, 54^. Rats are known to apply both reciprocity rules, direct and generalized reciprocity, while sharing food^37–40, 55–57^. Nevertheless, if partner-specific information for direct reciprocation can be used, this leads to higher cooperation levels than when rats interact with anonymous partners^39^.

There are several possible cognitive mechanisms explaining the pattern we found (reviewed in ref. 49). First, ‘symmetry-based reciprocity’ may underlie reciprocal allogrooming in rats. This is based on symmetrical traits, such as proximity or affiliation. Our experimental subjects showed more grooming of the same partner after they were allogroomed at enhanced rates following saltwater application. Because the same partner was used, potential confounds such as kinship or rank can be excluded. We cannot exclude an influence of proximity on the results, because in order to groom a partner, close proximity is required. However, there is evidence that rats do not take into account proximity, or copy a partner’s actions, when deciding to reciprocate food donations^40^: rats having cooperated in the same way by providing high or low-quality food were rewarded differently, according to the food quality they had provided. In these experiments, the proximity between focal rats and partners providing either high or low-quality food^40^, or between focal rats and partners providing food or no food^39^, was standardized, so proximity cannot explain reciprocation of food donations. The second possibility would be that rats use ‘calculated reciprocity’ by estimating payoffs based on received benefits and paid costs. This mechanism is not suggested by our data, however, because the number of given allogrooming bouts did not correlate with the number of grooming bouts received from the partner. The third cognitive mechanism proposed to explain reciprocal trading has been referred to as ‘attitudinal reciprocity’, assuming that animals form attitudes based on received help, on which decisions are based to help in return. This may seem a possible cognitive mechanism underlying reciprocal allogrooming in rats, which should be experimentally scrutinized in future studies.

The latency to donate the first food item to a partner appears to be a good indicator for the motivation to cooperate^38, 40, 56^. Consequently, we expected to find a shorter latency to start grooming cooperative than non-cooperative partners, but this did not occur. This may have resulted from the fact that focal rats and their partners were familiar to each other. In this study we had to use familiar partners, because rats who do not know each other are much less likely to allogroom (personal observations). Also in food-sharing experiments, rats that are familiar to each other reciprocate experienced help by increasing the number of food items they provide to their partner, but they do not start sooner to help cooperative than non-cooperative partners (M. Schweinfurth & M. Taborsky, unpublished data), which differs from the interaction between unfamiliar partners^38, 39, 56^.

In our experiment, dominant and subordinate partners had the same need to be groomed due to our standardized treatment; still, focal rats differed in their allogrooming propensity according to the partner’s relative rank, preferring to groom dominants. However, received allogrooming was reciprocated up and down the hierarchy, revealing that both mechanisms, rank effects and reciprocity, separately govern allogrooming rates. Our results indicate that allogrooming is not exclusively used for hygienic purposes, but that it also serves as an important mediator for social relationships. This may be similar in some ungulates, as they were shown to allogroom conspecifics even in virtually ectoparasite-free zoological parks^58^. Seyfarth’s model^25^ suggests that grooming should be unreciprocated if directed up the hierarchy, which does not apply to our rats: they reciprocally allogroomed both higher and lower ranked individuals. However, in accordance with Seyfarth’s model, they overall groomed dominant partners more often than subordinate ones, irrespective whether these individuals had been cooperative or non-cooperative before.

Elevated allogrooming rates towards dominant partners might be explained by coercion through dominant partners, as this has been suggested to influence the food provisioning tendency of Norway rats^55^. In wild Barbary macaques, a subordinate victim receives more aggression after a fight if the victim did not groom the dominant aggressor directly after a conflict^13^. Hence grooming a dominant in this species might result from coercion, but the victim could also use allogrooming as a pre-emptive appeasement gesture (cf. ref. 59). We did not observe any aggression between our rats that would suggest a role for coercion. Therefore, it seems more likely that allogrooming up the hierarchy might serve as an appeasement or ingratiation behaviour.

The steepness of the dominance hierarchy affects reciprocal allogrooming in female primates^26^: the steeper the hierarchy, the more unreciprocated help is expected, as observed for instance in bonobos^60^. In Norway rats, males form near-linear hierarchies based on fights, but females fight much less over dominance ranks^61^; they do have a dominance hierarchy^45^, but it is less steep than in males^62^. Hence, male rats might not reciprocate allogrooming to a similar degree as females due to their steeper hierarchy, which is an interesting question for future studies.

Hitherto, evidence for a reciprocal exchange of social services has been criticised as being based on artificial manipulative experiments^63, 64^ or on purely correlative studies^65^. In contrast, our simultaneous manipulation of the need to be groomed and of allogrooming behaviour induced a realistic and intuitive natural interaction, for which no prior conditioning is needed, which has been suggested for reciprocations in rats^66^. In a previous study of long-tailed macaques (*Macaca fascicularis*), allogrooming was manipulated by application of a sticky mixture of syrup and seeds^67^. Individuals that had been groomed supported groomers more often when these attacked another social partner than when they had not been allogroomed before. In contrast to using an attractive substance (syrup) to elicit allogrooming, we used a saltwater solution that is usually avoided by rats, in order to incur costs to the groomer. In addition, we manipulated the behaviour of the potential groomer to test for a causal relationship between received and given grooming. Finally, we measured the response of the experimental partner by enabling it to pay back the received favour in kind. Allogrooming seems to reflect the most frequently observed form of reciprocal cooperation in animals^19^. Our simple method allows manipulating allogrooming rates even in the field, which should help understanding the causal relationship of received and given allogrooming and allopreening in a wide range of animals.

## Electronic supplementary material

Supplementary materials
