# Supplementary material for: Experimental evidence for reciprocity in allogrooming among wild-type Norway rats
Source: Sci Rep. 2017 Jun 21;7:4010. doi: 10.1038/s41598-017-03841-3 (PMC5479849; doi:10.1038/s41598-017-03841-3)
Supplement: Supplementary file 1 — Supplementary materials [file 41598_2017_3841_MOESM1_ESM.doc]

**Supplementary materials**

**Experimental evidence for reciprocity in allogrooming among wild-type Norway rats**

Manon K. Schweinfurth^1,2^, Binia Stieger^1^ & Michael Taborsky^1^

^1^ Institute of Ecology and Evolution, University of Bern, Wohlenstr. 50a, CH-3032 Hinterkappelen, Switzerland

^2^ Correspondence: [manon.schweinfurth@iee.unibe.ch](mailto:manon.schweinfurth@iee.unibe.ch)

Rats do not prefer salt water

Rats living in groups (n= 12) of 4 to 5 female cage mates were provided with two bottles (600ml each) simultaneously for 24 hours. One bottle contained tap water, the other salt water of the same concentration as used in our allogrooming experiment (250g salt/l). Rats drank more tap water than salt water (pairwise Wilcoxon test: V= 0, p< 0.001, fig.1), hence the latter was clearly non-preferred.

| 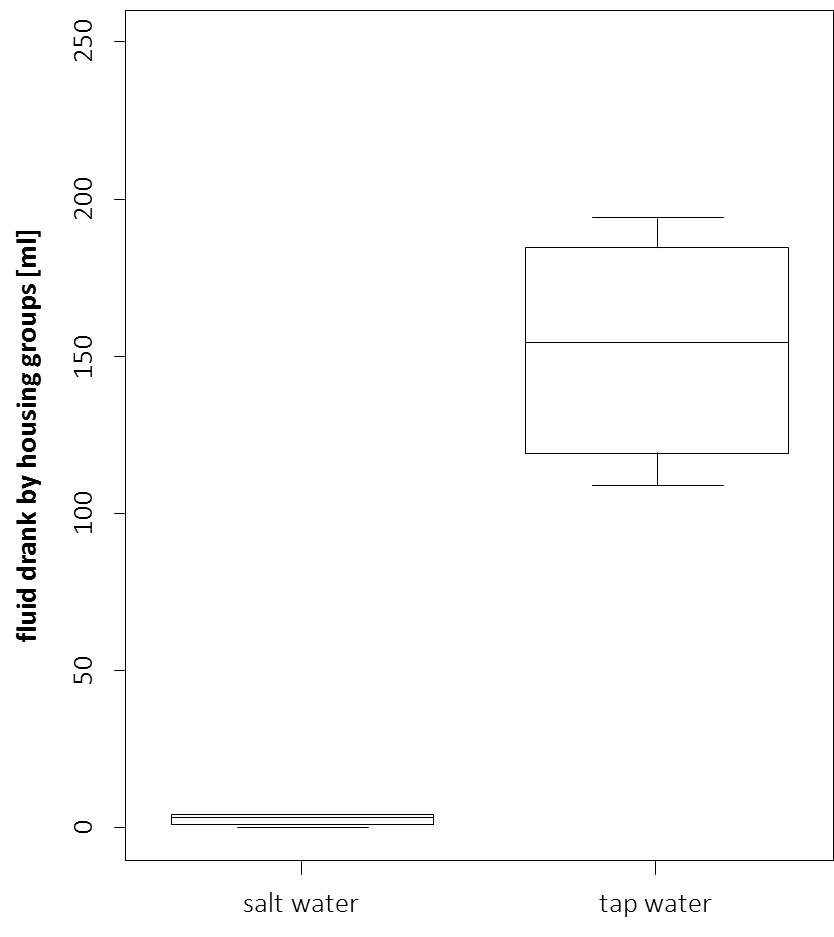 |
| --- |
| **Figure 1: Comparison of fluid consumed by rats within 24 hours when having the choice between tap water and salt water.** Rats drank almost exclusively tap water. Boxplots are shown with median with second and third quartiles in the box and first and fourth quartile of the data indicated by the upper and lower lines.s |

| 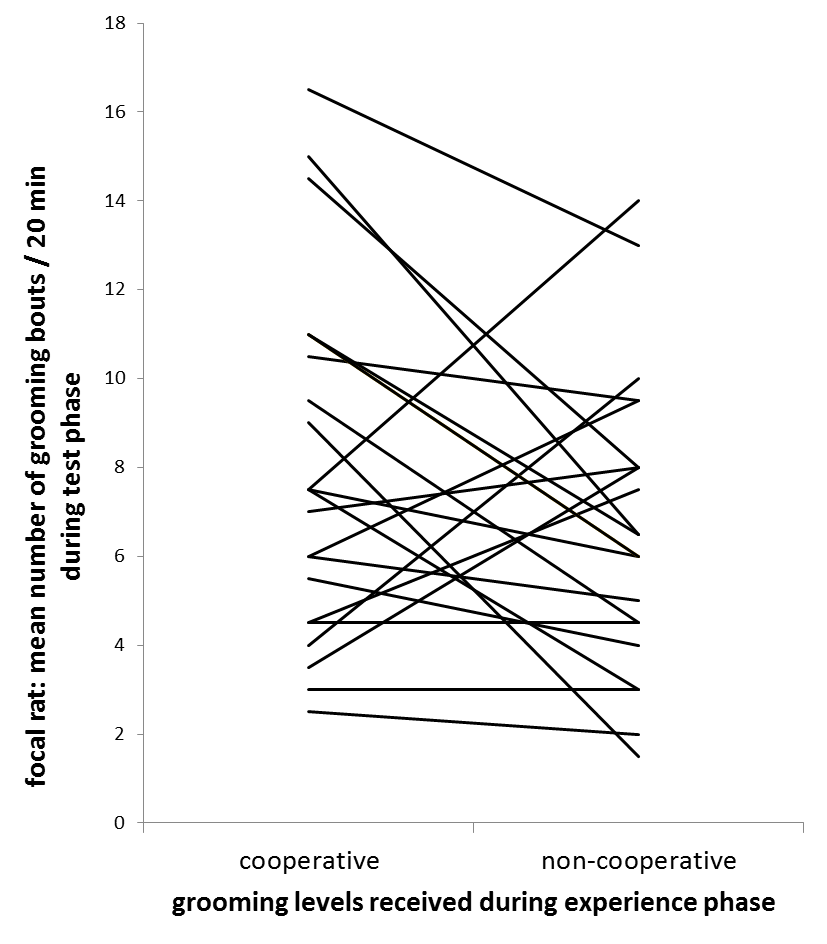 |
| --- |
| **Figure 2: Comparison of grooming bouts of focal rats towards their previously experienced cooperative and non-cooperative partners.** |

| 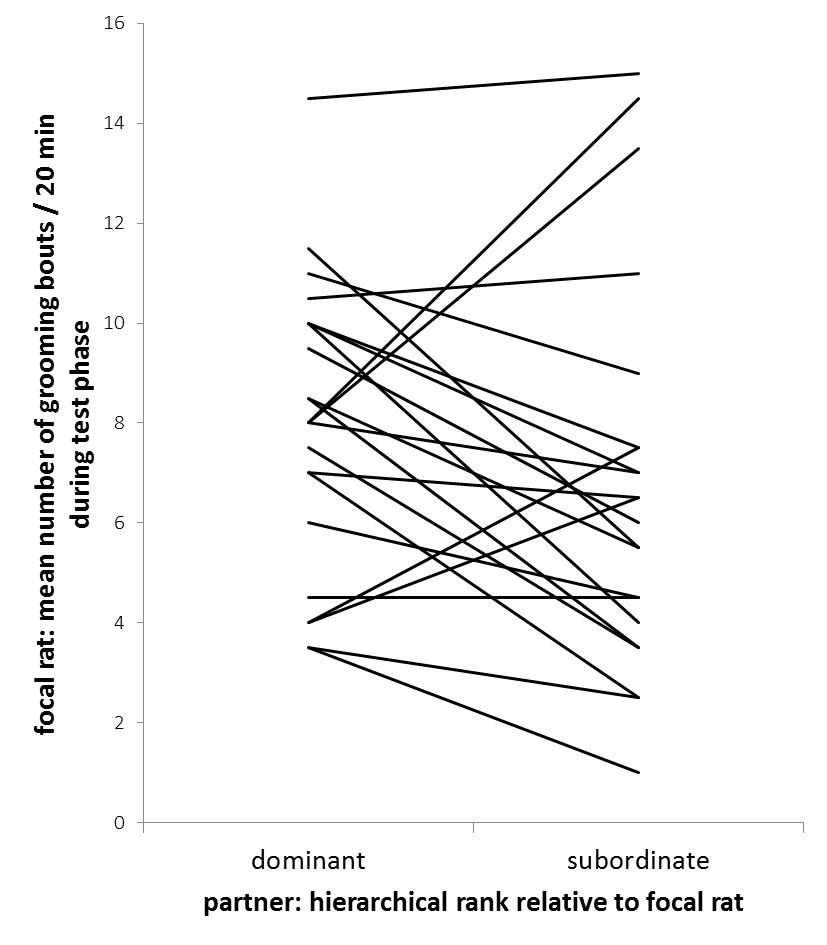 |
| --- |
| **Figure 3: Comparison of grooming bouts of focal rats towards their dominant and subordinate partners.** |

| 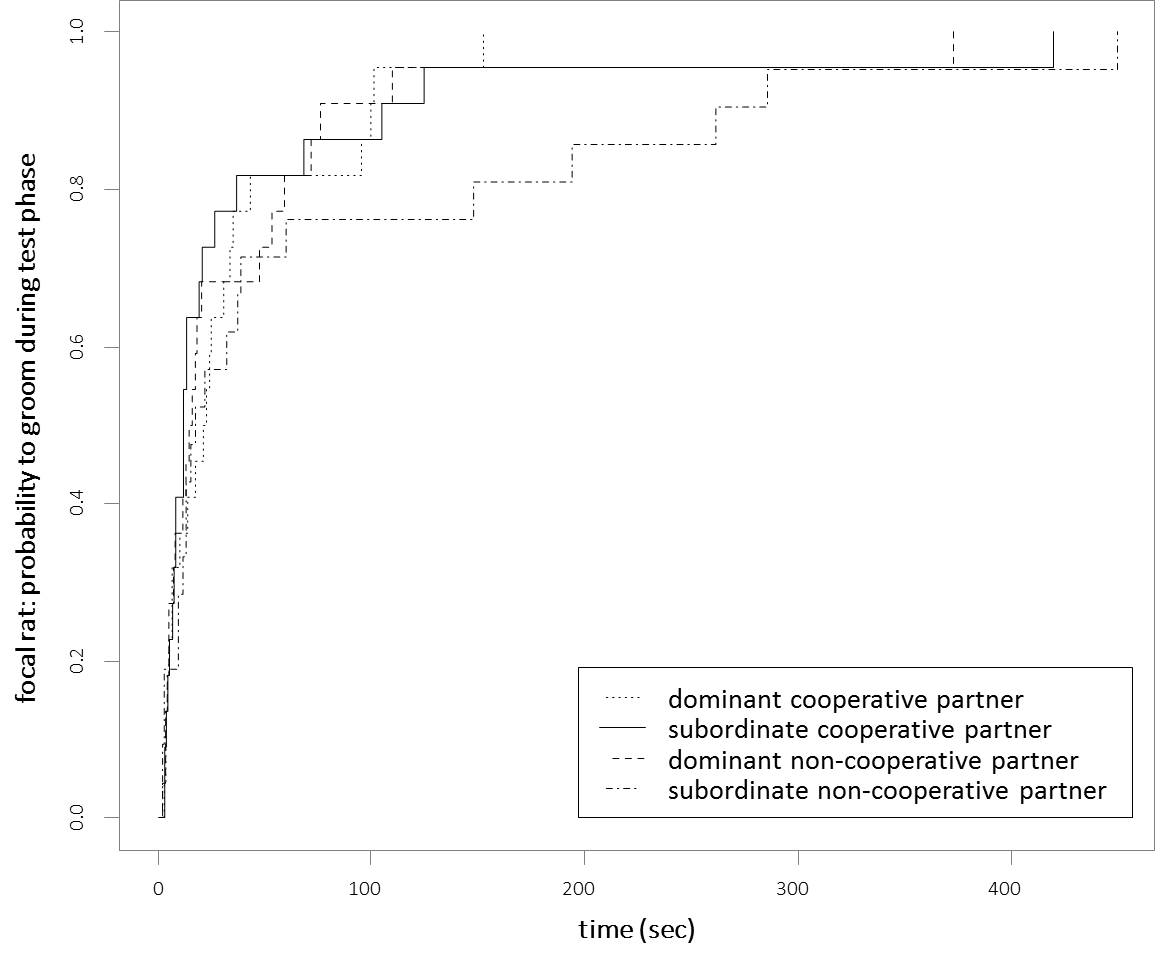 |
| --- |
| **Figure 4: Probability to start allogrooming the respective partners.**  The focal individuals’ latency to start allogrooming their respective partners neither differed between previously cooperative and non-cooperative partners, nor between dominant and subordinate partners. The cumulative probabilities of occurrence are shown. |
